# The Emerging Role of the Interactions between Circular RNAs and RNA-binding Proteins in Common Human Cancers

**DOI:** 10.7150/jca.58182

**Published:** 2021-06-26

**Authors:** Meng-Ping Jiang, Wen-Xiu Xu, Jun-Chen Hou, Qi Xu, Dan-Dan Wang, Jin-Hai Tang

**Affiliations:** Department of General Surgery, the First Affiliated Hospital of Nanjing Medical University, Nanjing, Jiangsu, China.

**Keywords:** circular RNAs (circRNAs), RNA binding proteins (RBPs), biogenesis, transcription, cancers

## Abstract

Circular RNAs (circRNAs) are a unique family of noncoding RNAs that could regulate multiple biological processes, which play a crucial role in carcinogenesis, progression and chemotherapy resistance of cancers. Growing studies have demonstrated that circRNAs act as novel biomarkers and therapeutic targets for cancers by sponging microRNAs (miRNAs). Up to date, another function of circRNAs, combining with RNA-binding proteins (RBPs), was uncovered. However, there is limit studies illustrating the underlying mechanism of circRNAs-RBPs interactions, as well as showing its roles in diverse types of cancers. In this review, we collected the biogenesis, properties of circRNAs, and then synthesize the connection between circRNAs and RBPs, and try to clarify its molecular mechanisms involving in the pathogenesis and progression of several common cancers, aiming to provide a brand-new insight to the prognosis and treatment strategy for cancers.

## Introduction

Circular RNAs (CircRNAs), with covalently closed loop structure, are a vital type of noncoding RNAs and have attracted plenty of researchers' attention in recent years. Actually, the incidental discovery of the first circRNAs, as a class of infectious plant viroids, could date back to as early as the 1970s 1. Owing to remarkable transition from traditional transcriptome analyses to accurate sequencing and bioinformatics, a large amount of circRNAs have widely identified in eukaryotic cells but viewed as transcription byproducts without function 2. Dramatically, it was first proposed that circRNAs functioned as miRNA sponge to regulate gene expression at post-transcriptional level 3, 4. Afterwards, emerging researches revealed other essential roles of circRNAs in diverse biology process: binding partners of RNA binding proteins (RBPs), regulators of transcription, and templates for translated protein 5-7. Nowadays, it has been found that abundant circRNAs to a large extent participate in development of human diseases, especially cancer, promoting to increase researches on underlying mechanism and providing potential candidates for effective disease diagnosis and treatment.

Although circRNAs generally act as a miRNA sponge to perform biological function, studies have shown circRNAs are involved in diverse pathological processes by binding to RBPs 7. RBPs are a class of proteins containing RNA-binding domains that integrate with and control target RNAs at the post-transcriptional level, including transcription, splicing, stabilization, localization, translation, translation 8. Previous studies have shown that hundreds of RBPs identified in the human genome are widely expressed in tissues, some of which exhibit aberrant expression under disease conditions 9.

With updated techniques like RNA pull-down and RNA immunoprecipitation (RIP), the physical interactions between circRNAs and RBPs are recognized and play an irreplaceable role in human pathogeneses, particularly in progression of tumors. Nevertheless, the number of reviews related to circRNAs-RBPs complexes and detailed mechanism is limited 7, 10.

In our review, we aim to summarize the intimate relationship between circRNAs and RBPs, meanwhile we focus on the influence of circRNAs-RBPs interactions in several common types of tumors, providing new sight into diagnostic and therapeutic tools for incurable human cancers in the future.

## The biogenesis of circRNAs

**Figure 1:**
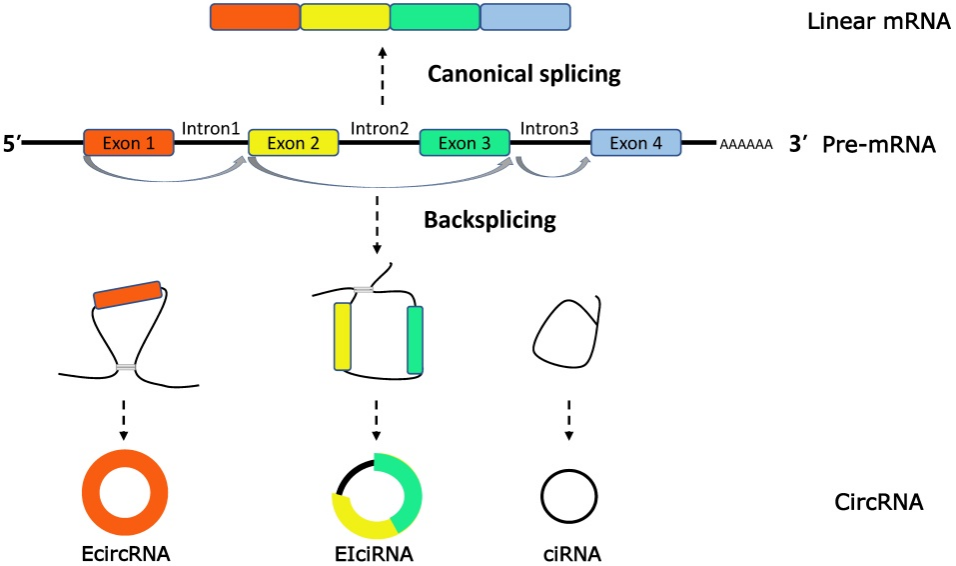
** CircRNAs biogenesis.** CircRNAs are generated in the process of splicing of pre-mRNA and compete with the counterparts, linear mRNA. CircRNAs are generally classified into three types (1) exonic RNAs (EcircRNAs), generated by exons only. (2) EIciRNAs, generated by introns 'retained' between the exons. (3) circular intronic RNAs (ciRNAs), generated by two or more connected introns.

Extensive studies on circRNAs have revealed that circRNAs are characterized with a ring covalently bound by a 5′ cap and 3′ poly (A) tails, produced by precursor-mRNA (pre-mRNA) back-splicing, which is distinct from the mature mRNAs generated by pre-mRNA through canonical or alternative splicings 11, 12. On the basis of the source of internal sequence, circRNAs can be classified into three categories: exonic circRNAs (EcircRNAs), intronic circRNAs (ciRNAs) and exon-intron circRNAs (EIciRNAs) 13 (**Figure 1**).

By collecting published researches, circRNA biogenesis have been demonstrated as a complex process regulated by three main biological regulatory mechanisms. As we concluded: 1) intronic repeat sequences. For example, the intronic repeats, in the Sry circular RNA biogenesis model, may base pair to one another, bringing the splice sites into close proximity to facilitate backsplicing 14. 2) Exon skipping and resplicing of the lariat RNA. Thorough mechanistic evidence was shown that a large lariat containing the skipped exon, followed by exons skipping during alternative splicing, is a common intermediate step before the production of a circular RNA 15. 3) RNA-binding proteins. Recently, it was reported that trans-acting splicing factors, such as hnRNPs and SR proteins, act to co-regulate pre-mRNA splicing patterns through site-specific binding to target RNAs 16.

Taken together, although there is increasing studies focusing on the formation of circRNAs at gene level, more detailed molecular mechanisms still need to be explore.

## The properties of circRNAs

Increasing studies have manifested that circRNAs have some predominant properties. First of all, the high stability of circRMAs has been widely acknowledged, mainly attributing to the covalent looping structure and resistance against RNase R 17. There is supporting evidence showing a longer average half-life of circRNAs, over 48 h, in comparison to the average 10 h linear RNA counterparts 18.

Followingly, circRNA is highly abundant in human tissues and cells. Thanks to RNA deep sequencing and computational algorithms, researchers have managed to identify over 25,000 presumptive circular RNAs stemed from more than 15% of encoding gene transcripts in human fibroblasts 11. In general, circRNAs still maintain a low expression as comparing to their host mRNAs.

Another characteristic of circRNAs is specificity in various tissues and developmental stage. For instance, an early study has demonstrated a tissue-specifically expression of circRNAs, with an enrichment in brain tissues 19. In virtue of circRNA sequencing, most of about 10,000 noval circRNAs are development-specific expressed in preimplantation human embryos 20.

Evolutionary conservation is a crucial property for circRNAs. For example, Xia et al. found approximately 700 homologous circRNAs between mouse and fetal human tissues, especially in brain 21. In addition, a mass of circRNAs has been identified in fungi, plants, and protists, indicating the feature of evolutionarily conserved circRNAs 22.

## The connection between circRNAs and RNA-binding proteins

As one of circRNAs' functions, previous bioinformatic analyses suggested that circRNAs were predicted to own abundant binding motifs for proteins, allowing circRNAs to combine with proteins, including RBPs. In recent studies, investigators are trying to uncover concrete contact patterns for circRNAs-proteins interactions, especially the connection between circRNAs and RBPs.

Firstly, accumulating studies have shown the existence and importance of RBPs participating in the formation of circRNAs *in vivo* or *in vitro* models. The most representative example is the RNA binding protein QKI, one of the members of the STAR family of KH domain-containing RNA binding proteins, was identified as a pre-mRNA splicing factor and a chief modulator of circRNA biogenesis 23, 24. Previously, it has been shown that QKI enhance circRNA formation via pre-mRNAs splicing regulation through binding to recognition elements within introns, in the vicinity of the circRNA-forming splice sites, and promoting circRNA-forming exons into close proximity 25. Lately, estrogen receptor α (ERα), a protein associated with progression of hepatocellular carcinoma (HCC), was found to directly bind to the 5′ promoter region of its host gene SMG1, thereby suppressing the circular RNA-SMG1.72 expression 26. In recent years, many RBPs, such as RNA helicase DHX9 27, RBM3 28, NF90/NF110 29, MBL 30, have been shown to a substantial contribution to the regulation of circRNA production.

While RBPs are able to regulate circRNAs biogenesis, circRNAs can relatively modulate the expression of RNA-binding proteins. A canonical example involves the tumor suppressor gene TP53-coded protein, p53 acts as a RBP to directly interact with circRNAs, which exerts joint function in the occurrence and progression of tumors 31. Additionally, it was shown that the expression of p53 can be regulated by some circRNAs. Among them, Circ-DNMT1 was reported to regulate p53 transcriptional activity by forming a heterogeneous nuclear ribonucleoprotein and translocating into the nucleus via the interaction with p53 itself and the other AUF1 32. A growing number of studies have indicated that other circRNAs are involved in the process of RBP transcription via a direct or indirect manner, including Circ-DNMT1 32, circ-Ccnb1 33, circ-MDM2 34, and so on.

Moreover, RBPs are more likely to directly or indirectly participate in circRNAs-mediated transcription. CircRNA function is also elicited by regulating the related gene transcription and gene expression. Moreover, it was evident that EIciRNAs hold U1 snRNP through interaction with U1 snRNA, and then further bind to Pol II transcription complex at the promoters of parental genes to enhance gene transcription 35. Additional support for an important role of RBPs on circRNAs modulating gene expression comes from bioinformatics and functional studies of HuR. It has extensively been shown to affect selectively the expression of certain mRNAs at similar post-transcriptional levels by binding to circ-PABPN1 rather than PABPN1 mRNA 36. Further studies have revealed that RBPs play a vital role in the transcription regulation of circRNAs.

Furthermore, increasing lines of evidence suggest that some RBPs are capable of facilitating translation of circRNAs. According to previous studies, N6-methyladenosine (m6A) located on circRNAs seems to a main mechanism responsible for the regulation of circRNA translation. The m6A is an adenosine methylation modification of RNA bases that can promote the efficient initiation of translation from circRNAs 37. Therefore, with the assistant of two RBPs, m6A demethylase FTO and adenosine methyltransferase METTL3/14, we could affect the translation efficiency through selectively altering m6A modification. In addition, different from linear mRNA translation, m6A-initiated circRNA translation needs to be initiated by protein factors, such as eukaryotic translation initiation factor eIF4G2 and m6A reader YTHDF3 38. To sum up, RBPs are essential regulators of the circRNAs translation.

## CircRNAs-RBPs interaction and cancers

Cancer is a common public health problem and the treatment of tumor and its internal mechanism are the hotpots of medical research. And, a large number of researchers have identified extensive circRNAs functioning as miRNAs sponge, emphasizing its important role in cancer development. Additionally, circRNAs-RBPs interaction is emerging as a new mechanism in several hallmarks of cancer, such as cell death and survival, invasion, and metastasis. To date, only a few reports have also shown that circRNAs may associate with specific proteins to exert important functions in cancer. Next, we mainly summarize some significant associations between RBPs and circRNAs in the following several types of tumors (**Table 1**).

### Breast cancer

Breast cancer (BC) is the most common female cancer worldwide and one of the leading cause of cancer-related death among women 39. Early breast cancer can be better improved by precise and individualized treatment, largely due to comprehensive therapies, including surgery, radiotherapy, chemotherapy and endocrinotherapy and targeted therapy. Despite this, treatment for advanced breast cancer remains a huge challenge, with a five-year survival rate of 26% 40. Therefore, valuable biomarkers deserve further exploration for the early detection, treatment and prognosis for BC.

CircRNAs have been proposed to have a key role in BC development through interacting with RNA binding proteins. Circ Foxo3 was a classic candidate that had a significant low expression in BC tumors and cancer cells as comparing to normal tissue and cells, respectively. Previously, it was reported that poly-ubiquitination function of MDM2 protein was essential in the degradation of p53 and Foxo3 41. Further study indicated that circFOXO3 directly interacted with p53 and MDM2, providing a platform for p53 degradation in MDM2-mediated manner. Of note, Foxo3 was identified to compete with p53 for binding circFOXO3, but up-regulated circFOXO3 had a higher affinity for p53, leaving FOXO3 activate downstream Puma-mediated apoptosis 42.

**Figure 2:**
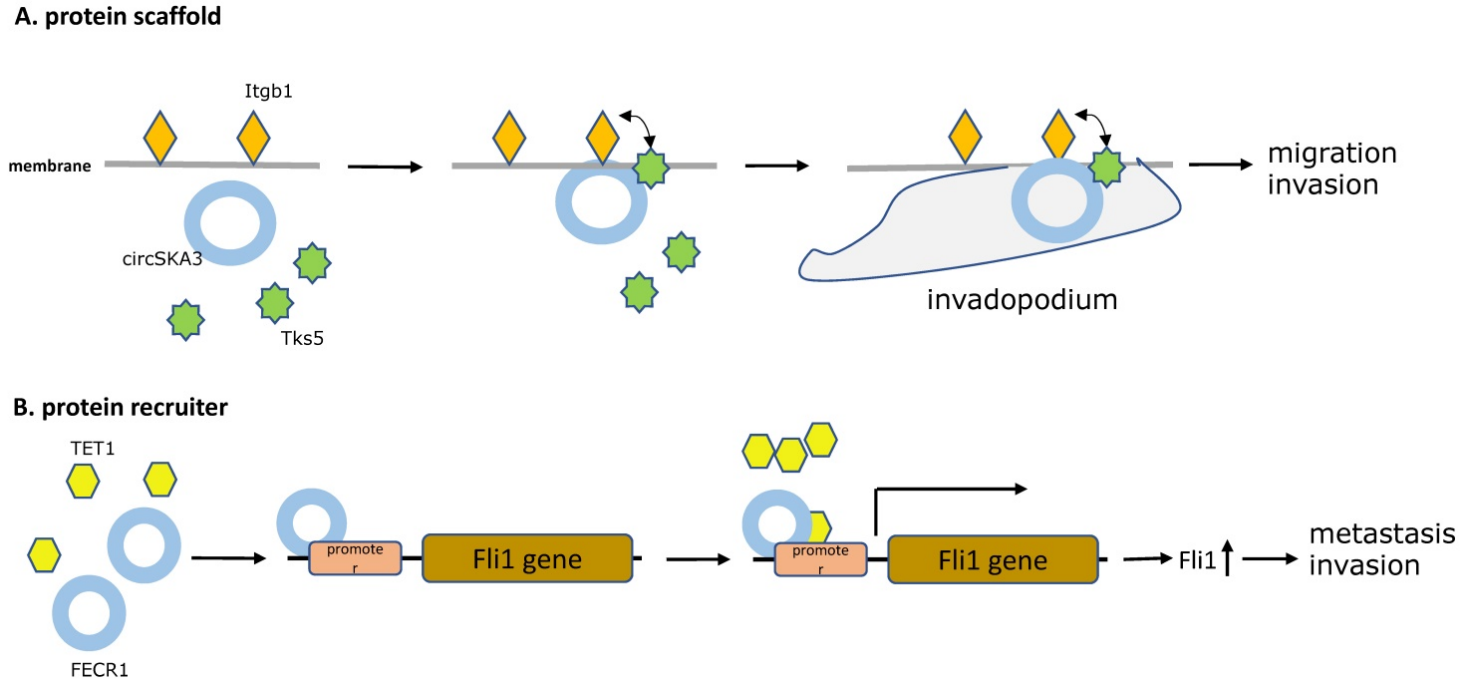

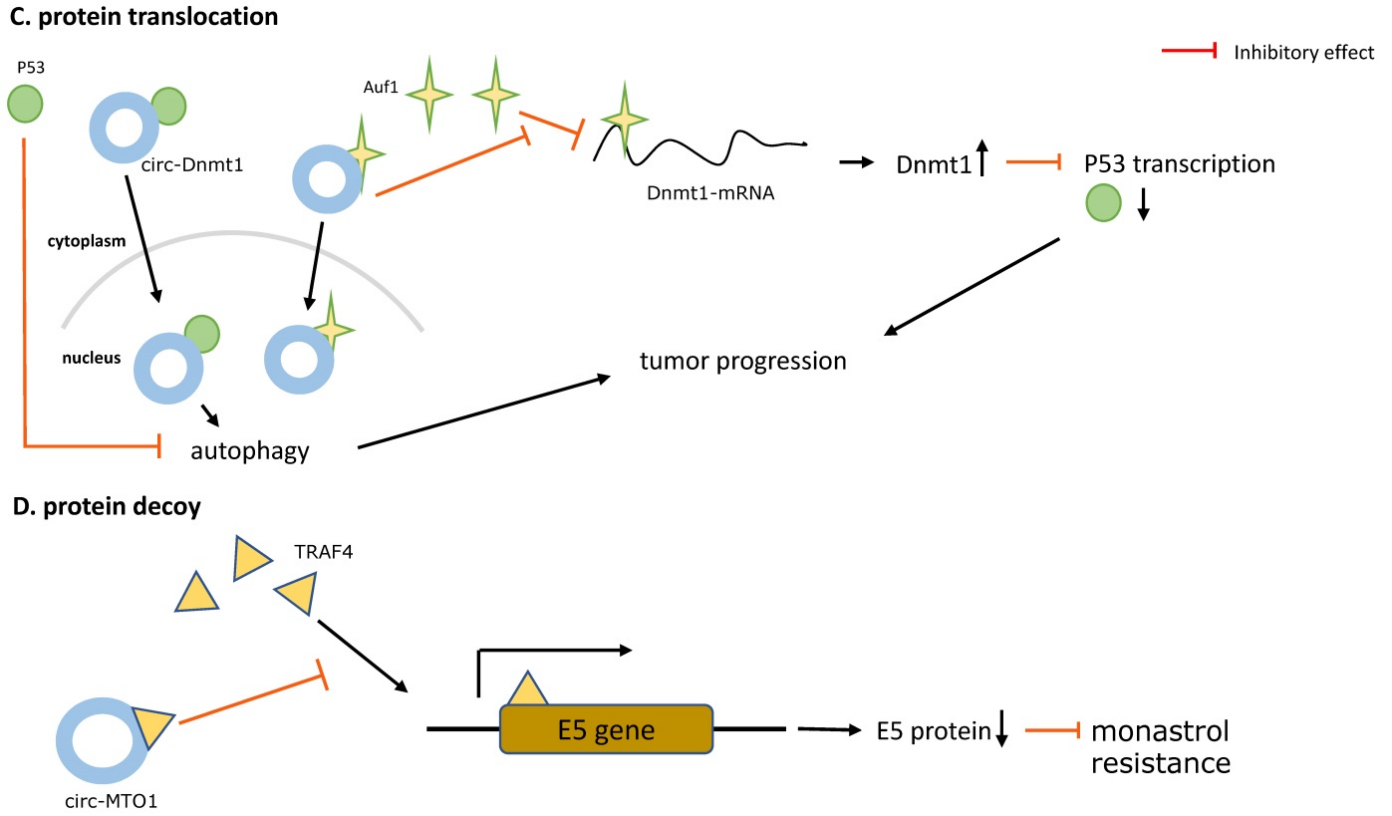

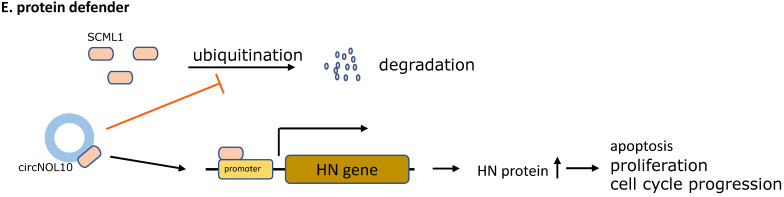
**The five main types of CircRNAs-RBPs interactions. A.** Protein scaffold.CircSKA3 binds to Tks5 and integrin b1, and serves as a protein scaffold for interaction of them on the cell membrane, inducing the production of invadopodia in breast cancer. **B.** Protein recruiter. FECR1 attaches to the parental gene FLI1 promoter and recruiters for TET1 demethylase to demethylate the promoter, stimulating oncogene FLI1 transcription to enhance tumor metastasis and invasion. **C.** Protein translocation.Circ-Dnmt1 promotes translocation of p53 and AUF1 from cytoplasm to nucleus, which enhances cellular autophagy and reduces p53 expression in the cytoplasm, thereby accelerating tumor progression. **D.** Protein decoy. CircRNA-MTO1 combines with TRAF4 and blocks its positive regulation of E5 translation, and finally reverses monastrol resistance. **E.** Protein defender. CircNOL10 physically connects with SCML1 and protects SCML1 from degradation induced by ubiquitination, thus the increased SCML1 expression activating transcription of the HN polypeptide family related to apoptosis, proliferation, and cell cycle progression in lung cancer.

A similar example, by microarray analysis, overexpressed circSKA3 was found to promote breast cancer cell migration and invasion in breast cancer cells and human breast cancer tissues. Mechanistically, providing evidence showed that the circSKA3 induced the formation of invadopodium to promote tumor progression by directly complexing with Tks5 and integrin b1, which was detected by immunoprecipitation of cell lysates 43. As in the previous article, Itgb1 is known as a kind of cell-surface receptor involved in the process of cell adhesion, detachment, and migration 44. Tks5 is an adaptor protein and its phosphorylation is critical for invadopodium formation 45. Notably, William Du et al. demonstrated that circSKA3 functioned as protein scaffolds and provided a bridge for Tks5 and integrin b1 on the cell membrane, which is the key complex in formation of invadopodia (**Figure 2A**).

In an another study in 2018, a novel circular RNA FECR1 derived from FLI1 exons, was illustrated to have a positive correlation with tumor invasion in breast cancer cell lines, suggesting its role as an oncogenic driver in metastasis of breast cancer. Furthermore, a novel epigenetic pathway was discovered that FECR1 could bind to the parental gene FLI1 promoter in cis and acts as a protein recruiter for TET1 demethylase which demethylated the promoter CpG islands, thereby activating transcription of the oncogene FLI1 to promote tumor metastasis 46 (**Figure 2B**).

Additionally, another oncogenic circRNA in breast cancer is circ-Dnmt1. Du et al. used microarrays analysis to identify circ-Dnmt1 upregulated in BC cell lines and tumors and that is link to breast cancer progression. Of note, circ-Dnmt1 utilized an autophagy mechanism to inhibiting cellular senescence and enhancing cell proliferation, survival, and tumor growth, which was induced by nuclear translocation of p53 and AUF1 via circ-Dnmt1 directly binding to its two oncogenic protein partners. Nuclear translocation of p53 enhanced cellular autophagy, in contrast to p53 in the cytoplasm, while the AU-rich RNA-binding protein AUF1 was transported into the nucleus increasing Dnmt1 translation and thus inhibiting p53 transcription 32 (**Figure 2C**).

According to another study published in 2018, a circRNA called circRNA-MTO1 was expressed at abnormally low levels in monastrol resistant cells and negatively regulated cell viability and monastrol resistance. In order to uncover the mechanism of circRNA-MTO1, Liu et al. used mass spectrometry and RNA-pull down assay to confirm the direct interactions between circRNA-MTO1 and tumor necrosis factor receptor associated factor 4 (TRAF4). TRAF4 is an (A+U)-rich elements (AREs)-binding protein, and can interact with ARE areas and functions as a potential oncogenic protein due to the high expression levels in human carcinomas 47, 48. Collectively, the findings of this study indicated that overexpressed circRNA-MTO1 could bind to TRAF4, blocking the TRAF4-mediated E5 translation and finally inhibiting viability and reversing monastrol resistance 49 (**Figure 2D**).

### Hepatocellular carcinoma

According to World Health Organization statistics, hepatocellular carcinoma (HCC) is the fourth most prevalent cause of cancer-related deaths globally. Due to aggressive tumor biological characteristics, HCC patients suffer a high rate of mortality, with ~841,000 new cases and 782,000 deaths every year 39. Currently curative liver surgery benefits patients at early stage, but exerts only limited effects on advanced HCC patients. Thus, it is urgent to elucidate the mechanisms underlying the development of HCC and explore the effective biomarker for early diagnosis and prognosis.

Accumulating evidence suggests that both of circRNAs and some certain RBPs have a crucial role in HCC.TIP60 is a classic cancer-related RNA-binding protein, contributing to tumorigenesis, mesothelioma malignance, cancer growth in various types of cancer 50-52. The main oncogenic effects of TIP60 appeared to trigger target gene transcription through governing histone acylation modification 53. To explore the role of TIP60 in liver cancer, Wang et al. used ChIP and FISH assays to indicate that TIP60 was recruited by circRHOT1 to combine with the NR2F6 promoter, and next actively recruited other components of NuA4 complex to finally enhance target gene NR2F6 expressions, resulting in suppressing HCC development and progression. Of them, circRHOT1 was also illustrated as a potential prognosis biomarker for HCC, depending on that patients' with high circRHOT1 was more likely related with poor prognosis 54.

Although FMRP serves as a RBP and exerts its function of regulating translation of target mRNAs mostly studied in the nervous system 55, its expression appeared to be significantly low in HCC 56. In the previous published study, circZKSCAN1 was confirmed to interact with FMRP and prevent tumor development by inhibiting cell stemness, proliferation, and metastasis in HCC. Mechanistical results indicated that the combination of circZKSCAN1 and FMRP sequestered FMRP from binding to β-catenin-binding protein-cell cycle and apoptosis regulator 1 (CCAR1) mRNA, which induced tumor quiescence by blocking the Wnt/β-catenin signaling pathway 57.

It was recently reported that cIARS appeared to be ectopic expression by using RNA-seq during sorafenib (SF) treatment in HCC cells. Functional experimental analysis suggested that cIARS was demonstrated as a positive regulator of SF-induced ferroptosis through activating autophagy and ferritinophagy. By RNA pulldown and RNA EMSA assays, the authors validated ALKBH5 as a potential interacting protein of cIARS, whose effect on autophagy regulation is reflected in a variety of tumor cells 58, 59. In this study, cIARS was proven to involve in SF-induced ferroptosis by bounding to its protein couple ALKBH5 and abolishing the autophagy inhibitor role of ALKBH5 in HCC cells 60.

As a recent research indicated, circBACH1 expression level was increased specifically in HCC tissues and cell lines. An analysis of clinical data revealed that the HCC patients with high circBACH1 expression may have poor prognosis. Moreover, based on cell function experiments, circBACH1 was confirmed to facilitate cell proliferation by negatively modulating p27 expression. In addition, RIP assays, pull‐down assays and EMSAs was performed to identify HuR as circBACH1 binding partner, which had been found to target p27 mRNA and repress its translation 61. P27, belongs to cyclin‐dependent kinase inhibitor family, is a tumor suppressor responsible for cell cycle arrest at G1‐S stage 62. In brief, Liu et al. illustrated that HuR could combined with circBACH1 in the nucleus and transported into the cytoplasm, where HuR inhibited p27 expression at translational levels 63.

Circ-LRIG3, an overexpressed nuclear circRNA in HCC, was found to enhance HCC tumorigenicity and progression. In mechanism, Sun et al. indicated that Circ-LRIG3 acted as a protein scaffold for EZH2 and STAT3 and activated STAT3 signaling through methylating and phosphorylating STAT3 induced by EZH2. In turn, a positive feedback loop was uncovered that activated STAT3 could directly interact with circ-LRIG3 promoter to facilitate circ-LRIG3 transcription and increase its expression 64. EZH2 is a notable oncogenic RBP and was previously reported to directly bind to another tumor suppressor, circ-ADD3, in HCC. Other than circ-LRIG3-EZH2-STAT3 ternary complex, EZH2 was able to bind to CDK1 in the presence of circ-ADD3 and increased phosphorylation and ubiquitination of EZH2, resulting in EZH2 degradation. Subsequently, low EZH2 protein expression weakens its tumor metastasis-promoting effect in HCC 38.

### Gastric cancer

Gastric cancer (GC), as one of the most frequently occurring malignancy, has become the third major cause of cancer deaths in China 65. Although rapid advances in diagnosis and treatment had partly improved patient outcomes, the 5-year survival rate is still quite poor, as shown in global cancer statistics 65. Therefore, it is imperative to seek novel biomarkers and significative therapeutic target for diagnosis and treatment for GC.

Mounting data suggests that circRNAs are related to gastric cancer (GC) development, but only a few studies uncover their mechanism of action in GC development, especially the circRNAs-RBPs interaction.

As a significant cancer-related circRNA, CircAGO2 is up-regulated in human cancer tissues, such as gastric cancer, colon cancer, prostate cancer, and is closely relevant to poor prognosis of tumor sufferers. The physically binding CircAGO2 to HuR was confirmed by RNA pull-down and western blot assays, which played an important role in promoting gastric cancer progression. Further studies discovered that CircAGO2-HuR complex enhanced the translocation of HuR from nucleus to cytoplasm, where HuR directionally tethered to the 3'-UTR regions of target oncogenes and followingly prevented AGO2 from binding and blocked downstream AGO2/miRNA signal pathway 66.

According to functional assays, Fang et al. indicated a negative correlation between the expression of circFAT1 (e2) and GC cell proliferation, migration and invasion. Besides, Y-box binding protein-1 (YBX1), a RBP known as a potential biomarker in GC diagnosis, has been found to positively regulate gastric cancer cells migration 67, 68. Moreover, it was demonstrated that circFAT1 (e2) may directly interact with YBX1 in the nucleus and initiate its tumor suppressive effect by performing the online catRAPID analysis and circRIP assay 69.

Another pathway associated with gastric cancer progression is the CircMRPS35 interacting with histone acetyltransferase KAT7.CircMRPS35, a noval circRNA, was first found in gastric cancer tissues with a low expression level, selected from differential expression profiles of circRNAs in human gastric cancer and adjacent normal tissues by RNA-seq. It was proposed that overexpression CircMRPS35 could specifically bind to FOXO1/3a promoter regions and hence activate the transcription of FOXO1/3a via H4K5 acetylation after recruitment of KAT7.In turn, the activation of FOXO1 positively stimulates the expression of its downstream genes p21, p27, PUMA and Bim, while activated FOXO3a upregulates E-cadherin and downregulated Twist1, eventually restraining cell invasion and metastasis in GC 70, 71. Therefore, we could hypothesize that CircMRPS35 is becoming a promising druggable target for antitumor treatment under the regulation of histone modification 72.

In addition, another new circRNA called Circ-HuR (hsa_circ_0049027) was shown to be remarkably down-regulated in gastric cancer tissue, whose high expression was related to inhibiting aggressive features such as the growth, invasion, and metastasis of gastric cancer cells. CCHC-type zinc finger nucleic acid binding protein (CNBP), known as a transcription factor, was previously proven its meaningful impact on malignant behavior of tumor cells 73, 74. Particularly, a proteomic analysis was performed to suggest the binding of circ-HuR to CNBP arrested the function of CNBP on facilitating HuR expression and suppress tumor progression 75.

### Lung cancer

Lung cancer (LC) is the most dominant cause of cancer-related death worldwide, mainly owing to highly reoccurrence and metastasis after therapy 76, 77. Non-small-cell lung cancer (NSCLC), representing 85% of the total lung cancer cases, has been considered as the most common histological kind of lung cancer, while small-cell lung cancer (SCLC) is known as the most lethal lung cancer with very low survival rates 78. To cut down LC deaths, scientists are trying to understand the detailed molecular mechanisms in LC in order to find more potential therapeutic strategies for LC.

Studies have suggested that circRNAs and related RBPs could serve as diagnostic or predictive biomarkers for lung cancer, and could provide new insights especially in NSCLC.

It was well recognized that circNOL10 was low-expressed in lung cancer tissues, as well as lung cancer cells, whereas circNOL10 upregulation contributed to repression of tumor development. Most notably, circNOL10 was shown to physically bind to the transcription factor SCML1, and subsequently increased the SCML1 expression, instead of inhibiting binding protein functions. Moreover, the circNOL10-SCML1 complex was able to restrain from SCML1 degradation induced by ubiquitination, thus up-regulated SCML1 further activated transcription of the HN polypeptide family associated with apoptosis, proliferation, and cell cycle progression in lung cancer 79 (**Figure 2E**).

A newly study verified that circ-SOX4 is positively correlation with CD133 expression and highly expressed in CD133+ lung cancer cells. Results from functional assays proved that downregulated circ-SOX4 restrained lung tumor-initiating cells (TICs) proliferation, self-renewal, migration and invasion. c-MYC, a pivotal RNA-binding protein, has been previously delineated involvement in NSCLC progression 80. In this study, circ-SOX4 upregulation was illustrated to enhance c-MYC expression via triggering the Wnt/β-catenin axis in NSCLC, while up-regulated c-MYC enabled circ-SOX4 to accelerate transcription by binding to circ-SOX4 promoter, thus forming a positive feedback 81.

G protein-coupled oestrogen receptor (GPER), a component of the G protein-coupled receptor (GPCR) family, has been regarded as a oncogenic driver to promote tumor initiation and development through YAP1/TEAD signalling in breast cancer 82, 83. Similarly, Shen et al. suggested that GPER enhanced cell growth of non-small cell lung cancer cells by circNOTCH1/m6A methylated NOTCH1 axis, whereby the physical interaction between circNOTCH1 and METTL14 played a compelling role in GPER downstream regulatory network. As a RBP, METTL14 is capable of undergoing target mRNAs m6A modifications and affecting its stability. Besides, circNOTCH1 served as endogenous competitive circRNA to compete for METTL14 binding. Due to the lack of METTL14, NOTCH1 mRNA appeared to be more stable and tended to be transcribed to high expression of NOTCH1, ultimately leading to initiate NOTCH1-mediated signal pathway 84.

### Glioma

Glioma is the most prevailing tumor of the central nervous system (CNS), accounting for 30% of all CNS tumors, of which glioblastoma multiforme (GBM) is regarded as most aggressive glioma 85. In spite of tremendous efforts and progress made in surgical operation, radiotherapies to prolong survival time, the prognosis of patients with GBM is still poor, with a median overall survival at approximately 15 months 86. Hence, elucidating the underlying mechanisms of glioma progression is warrant to screen more proper biomarkers and effective therapeutic measures for glioma.

Numerous circRNAs, along with its binding proteins, have been widely detected at aberrant expressions in normal tissue of central nervous system, including glioblastoma, emphasizing their potential roles as promising prognostic, diagnostic, and therapeutic molecules.

It was observed that circSMARCA5 expressed at low expression in glioblastoma multiforme GBM biopsies, and was negatively correlated with glioma's histological grade in affected subjects. Furthermore, circSMARCA5 could not only suppress the migration of glioblastoma cells, but also involve in VEGFA mRNA splicing and angiogenesis in glioblastoma multiforme 87, 88. To further explore the underlying molecular mechanism, a bioinformatics analysis revealed that circSMARCA5 harbored abundant binding sites for RNA binding proteins, strongly suggesting that the circRNA-RBP interaction was essential for performing the action of circSMARCA5. Among the RBPs, erine and arginine rich splicing factor 1 (SRSF1), a splicing factor, was predicted to bind circSMARCA5 and then disrupt splicing within GBM cells, participating in GBM pathogenesis. SRSF1 is an upregulated protein in several cancer and exhibits many biological function depending on its various downstream target genes of splicing pattern. On the one hand, overexpressed SRSF1 was able to promote the expression of PTBP1 and SRSF3, which were likely the positive regulators of GBM cells migration 87, 89. On the another hand, SRSF1 had an influence on VEGFA-mediated angiogenesis through affecting alternative splicing of VEGFA pre-mRNA 88. Therefore, further studies revealed that circSMARCA5 exerted its function in GBM by specially binding to SRSF1, makes it possible that circSMARCA5 works as GBM biomarker into clinical practice 87.

As one of the potential circRNA in glioma, CDR1as expression is greatly decreased in glioma as comparing to adjacent normal brain, which is positively correlation with patients outcomes. A latest article was firstly reported that CDR1as exerted its suppression effect on tumorigenesis by binding firmly to tumor suppressor p53 protein at core DNA-binding domain, rather than sponging miRNA, where is exactly essential for MDM2 interaction. Consequently, binding tightly to p53, CDR1as acted as a protein defender to protect p53 from ubiquitination and degradation in MDM2-depent manner, eventually repressing tumorigenesis of glioma 90.

## Clinical implication of the circRNAs-RBPs interactions

### Biomarker

In line with different circRNA-RBP interactions in different types of tumors, we find that the abnormally expressed circRNAs or RBPs in some tumors are closely related to the patients prognosis respectively, highlighting their potential for tumor biomarkers.

circRNA is a kind of promising candidates for predicting living status of tumor patients. In a study published in 2017, a cohort of 116 HCC patients with survival data and corresponding circRNA-MTO1 expression were collectively analyzed by Kaplan-Meier survival curve, revealing the positive correlation between circRNA-MTO1 expression and prognosis of HCC patients and thus suggesting its potential as a prognosis biomarker 91. Microarray analysis of HCC tumor tissues of 112 patients in another study indicated that downregulated circZKSCAN1 was associated with various HCC clinic pathologic features. Based on Kaplan-Meier survival analysis, HCC patients with high circZKSCAN1 expression level were likely to own a better overall and recurrence-free survival. It was demonstrated that circZKSCAN1 expression was an independent factor relating with overall survival and relapse-free survival rate of HCC patients, supported by univariate and multivariate analysis 57. In addition to the stability of the looping structure of circRNAs, its widespread distribution in body fluids makes it more possible to be used as a simple clinical indicator.

The other protagonist in our article, RBPs, have clinical significances as well, depending on differential expression of RBPs in tumor and normal tissues. One of the representatives for RBPs is YBX1, which has been recognized as a marker for tumor aggressiveness and poor prognosis in various cancers such as breast, ovary, and liver cancer 92-94. The researchers indicated ectopic YBX1 expression associated with shorter DFS and verified its role as a biomarker, shown as analysis of 94 patients who underwent surgery in advanced GC 95. More recently, deregulated ALKBH5 may be capable of predicting an unfavorable clinical outcome in NSLC, as patients with low ALKBH5 expression tended to survive shorter than those with low ALKBH5 expression 96.

However, in light of the detection of circRNA in fluid, it seems more applicable to clinical using than RBPs. In fact, more datas and experimental proof are needed to support their role of biomarker before being put into clinical setting.

## Therapeutic strategy

We can propose an effective therapeutic envision for cancer treatment through distinctively targeting circRNA-RBP axes we summarized in several tumors above.

When it comes to circRNAs, it can be classified as tumor suppressors and oncogenes. Some deregulated circRNAs in tumors are considered as tumor suppressors. Therefore, it is a promising targeted therapy that artificially synthesizing tumor suppressor circRNAs. Previously, the efficiency of synthetic circRNAs was proved to be significant for gastric and esophageal cancers therapy 97, 98, thus similar thoughts may also be broadly applicable to other type of cancers. For another, oncogenic circRNAs are overexpressed in tumors, inhibiting its expression levels may work as another therapeutic strategy. Recently, shRNA-based knockdown of FECR1 was confirmed to diminish recruitment for TET1 demethylase and inactivate transcription of FLI1, resulting in repression of breast tumor metastasis 46. Similar to significant circRNAs, some circRNA-binding proteins can become a therapeutic target by regulating the expression or activity of RBPs or modulating connection of the circRNA-RBP comlex. For example, MS-444 is an inhibitor of the oncogenic RNA-binding protein HuR, leading to tumor cell apoptosis in malignant glioma via affecting HuR ability in the RNA binding and trafficking 99. Besides, CMLD-2, another HuR inhibitor, was found to reduce cell viability and promote apoptosis in thyroid cancer by obstructing interaction between HuR and mRNA targets 100. Above results suggest that potential HuR-targeted therapy for cancers may be put into clinical practice. Additionally, TET1, which belongs to ten eleven translocation (Tet) family dioxygenases, was shown to be essential for DNA demethylation and enhanced target FLI1 gene transcription in BC 46. Interestingly, it was previously reported that Vitamin C served as a cofactor and increased TET activity in HCC cells through directly binding to the catalytic domain of TET proteins 101. Notably, the circRNAs -RBPs interaction, along with their downstream regulatory networks, is likely to be specific targets for treatment of cancers, providing us with more ideas for developing novel anti-tumor therapies. However, whether these findings exert a meaningful role in clinical setting still need further investigation.

## Conclusions and perspectives

Over the years, the number of studies on the function of circRNAs, as shown in cancer cell lines and models, dramatically increases to the peak, most of which outlines the role of circRNAs as miRNA sponge, proteins sponge, gene transcriptional regulator, a template for proteins coding. Among them, the ability of circRNAs to bind to proteins gained a growing attention, which was predicted by valid bioinformatics algorithms and confirmed by RIP, RNA pulldown and other binding assays. As previous studies suggested, circRNAs-RBPs axes made difference to tumorigenesis and aggressiveness of tumors. However, the understanding of circRNAs-RBPs interaction in diverse tumor types have not been fully disclosed. Thus, we not only intended to expound biogenesis and properties of circRNAs, elaborate the reciprocity of circRNAs and RBPs, but also summarize manifold circRNAs-RBPs relationships in several common tumors, such as breast cancer, hepatocellular carcinoma, gastric cancer, lung cancer and glioma.

A number of studies have revealed the relationship between circRNAs and RNA-related proteins, including RBP. In brief, a few proteins were involved in circRNAs biogenesis and inversely some circRNAs were associated with expression of proteins. On the other hand, a number of RNA-related proteins were competent in influencing the regulation of target genes expression medicated by circRNAs, directly or indirectly. Moreover, it was discovered that many proteins, like PES1 and METTL3/14, were capable of promoting specific circRNAs translated to proteins. Besides, circRNA-RBPs interactions also vary under different pathophysiological conditions. Although uncountable circRNAs in human tissues or cells have been recognized, there are many unsolved puzzles about more detailed connections between circRNAs and RBPs, possibly due to the lack of detection methods for their relationships in living cells. Surprisingly, it is understood that many emerging technologies for RNA-proteins interactions, such as a psoralen probe (PP)-based method 102, have been developed and expected to be extended to this field to solve this problem, aiming to make great progress in the field of circRNAs.

Here, we firstly roundly discussed the roles of circRNAs-RBPs connection in cancer, enumerating studies that might deepen our comprehension of how it modulates cancer malignant behaviors. In several universal tumors we mentioned, circRNAs were able to interact with relevant proteins by acting as protein scaffold, protein recruiter, protein translocation, protein decoy and protein defender, exerting a vital role in tumor cell proliferation, apoptosis, autophagy, migration, invasion, metastasis. Without a doubt, the pathways we reviewed underlined clinical application of circRNAs and RBPs as the prognosis biomarkers for cancer patients and provided a wealth of targets for oncotherapy, including circRNAs-RBPs complex and downstream regulators. M.Puttaraju and his colleague have shown the intron-exon array (PIE) method, which could be used to generate circRNA drugs 103. This synthetic circRNA inhibitor may be an important member of future targeted therapies for cancer. In addition, our results attempted to lay a firm foundation for researchers to explore more precise regulatory networks in tumors.

It is worth noting that one of the hot spots in the field of circRNA today is the discovery of circRNA enrichment in various body fluids, including plasma 104, serum 105 and saliva 106, as well as exosomes 107. This facilitates the detection of circRNAs and thus makes them strong candidates for biomarkers in early diagnosis of cancer. Additionally, Xinyi Wang et.al found that exosomes were involved in intercellular signal delivery and might provide a promising therapeutic target for cancer 108. But nowadays more researches on exosomes continue to deepen.

In conclusion, some circRNA-RBPs interactions widely exist in a variety of cells, not just in specific cells, but others only under certain pathophysiological situations. Therefore, wider and more comprehensive researches are urged to perform to understand this relationship, which is expected to become a novel research hotspot with clinical promise to benefit tumor patients.

## Figures and Tables

**Figure 3:**
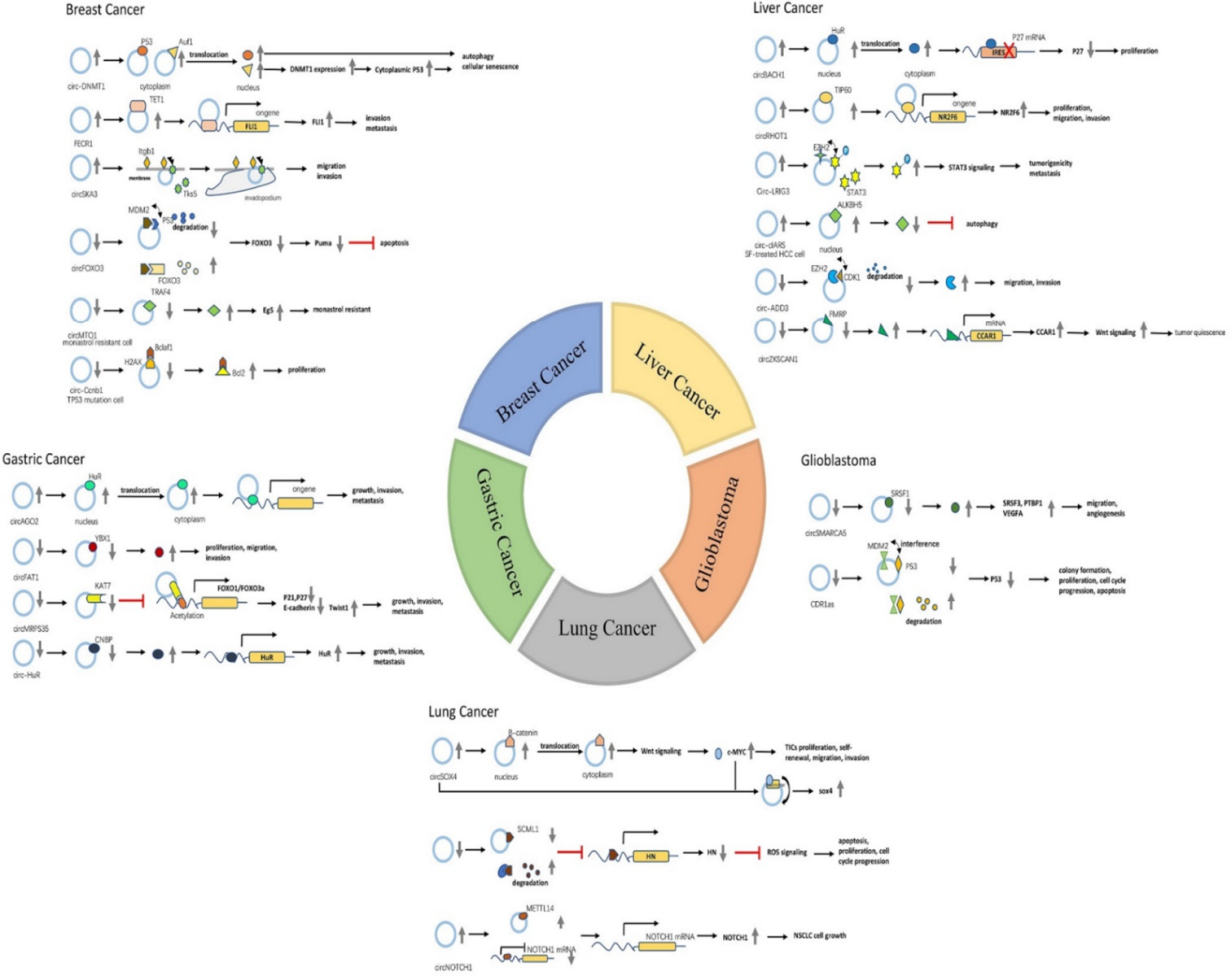
The detailed mechanism of CircRNAs-RBPs interactions in five common human cancers.

**Table 1:** Known circRNAs-RBPs interactions in several common types of tumors

Cancer	CircRNA	Dysregulation	Type of tissue/cell lines	RBPs	CircRNAs-RBPs interaction	Participation	Ref.
Breast cancer	circFOXO3	down	BC tissues and cells	MDM2,p53	protein scaffold	apoptosis	2017 42
	FECR1	up	BC tissues	TET1	protein recruiter	invasion, metastasis	2017 46
	circ-Ccnb1	down	BC cell lines	H2AX,p53; H2AX,Bcla1	protein scaffold	proliferation, apoptosis	2017 33
	circDNMT1	up	BC cell lines	p53,AUF1	protein translocation	autophagy mediated cell proliferation, survival, and tumor growth	2018 32
	circMTO1	down	monastrol-resistant cell lines	TRAF4	protein decoy	monastrol resistance	2018 49
	circSKA3	up	BC tissues and cells	Interinβ1,Tks5	protein scaffold	migration, invasion	2020 43
Liver cancer	circRHOT1	up	HCC tissues	TIP60	protein recruiter	proliferation, migration, invasion	2019 54
	circ-ADD3	down	HCC tissues	EZH2,CDK1	protein scaffold	migration, invasion, metastasis	2019 38
	circZKSCAN1	down	HCC tissues	FMRP	protein decoy	tumor quiescence	2019 57
	circ-cIARS	up	SF-treated HCC cells	ALKBH5	protein decoy	autophagy	2020 60
	circ-LRIG3	up	HCC tissues, cells and plasma	EZH2,STAT3	protein scaffold	tumorigenicity, metastasis	2020 64
	circBACH1	up	HCC tissues	HuR	protein translocation	proliferation	2020 63
Gastric cancer	circAGO2	up	GC tissues and cells	HuR	protein translocation	growth, invasion, metastasis	2019 66
	circFAT1(e2)	down	GC tissues and cells	YBX1	protein decoy	proliferation, migration, invasion	2019 69
	circMRPS35	down	GC tissues	KAT7	protein recruiter	growth, invasion, metastasis	2020 72
	circ-HuR	down	GC tissues and cells	CNBP	protein decoy	growth, invasion, metastasis	2020 75
Lung cancer	circNOL10	down	LC tissues	SCML1	protein defender	apoptosis, proliferation, cell cycle progression	2019 79
	circ-SOX4	up	CD133+ NSCLC cells	β-catenin, c-MYC	protein translocation, protein defender	TICs proliferation, self-renewal, migration, invasion	2020 81
	circNOTCH1	up	NSCLC cell lines	METTL14	protein decoy	NSCLC cell growth	2020 84
Glioblastoma	circSMARCA5	down	GBM tissues	SRSF1	protein decoy	migration, angiogenesis	2018 87, 2019 88
	CDR1as	down	GBM tissues	MDM2,p53	protein defender	colony formation, proliferation, cell cycle progression, apoptosis	2020 90

## Abbreviations

AUF1: AU-rich RNA-binding protein
BC: breast cancer
CCAR1: protein-cell cycle and apoptosis regulator 1
ChIP: chromatin immunoprecipitation
circRNAs: circular RNAs
ciRNAs: intronic circRNAs
CNBP: CCHC-type zinc finger nucleic acid binding protein
CNS: central nervous system
DHX9: DExH-box helicase 9
EcircRNAs: exonic circRNAs
EIciRNAs: exon-intron circRNAs
EMSA: electrophoretic mobility shift assay
FISH: fluorescence *in situ* hybridization
GBM: glioblastoma multiforme
GC: gastric cancer
GPCR: G protein-coupled receptor
HCC: hepatocellular carcinoma
hnRNPs: heterogeneous nuclear ribonucleoprotein
HuR: human antigen R
Itgb1: integrin b1
LC: lung cancer
MBL: muscleblind
MDM2: murine double minute 2
miRNAs: microRNAs
NF90/NF110: double-stranded RNA-binding domain containing immune factors
NSCLC: non-small-cell lung cancer
pre-mRNA: precursor-mRNA
QKI: Quaking
RBM3: RNA-binding protein 3
RBP: RNA binding protein
RIP: RNA immunoprecipitation
RNA-seq: RNA sequencing
SCLC: small-cell lung cancer
SF: sorafenib
snRNA: small nuclear RNA
snRNP: mall nuclear ribonucleoprotein
SR proteins: serine-arginine proteins
SRSF1: erine and arginine rich splicing factor 1
Tip60: 60 kDa Tat-interactive protein
TRAF4: tumor necrosis factor receptor associated factor 4
YBX1: Y-box binding protein-1
